# The Importance of Family and School Protective Factors in Preventing the Risk Behaviors of Youth

**DOI:** 10.3390/ijerph19031630

**Published:** 2022-01-31

**Authors:** Josipa Mihić, Martie Skinner, Miranda Novak, Martina Ferić, Valentina Kranželić

**Affiliations:** 1 Laboratory for Prevention Research, Department for Behavioral Disorders, Faculty of Education and Rehabilitation Sciences, University of Zagreb, 10000 Zagreb, Croatia; miranda.novak@erf.unizg.hr (M.N.); martina.feric@erf.unizg.hr (M.F.); valentina.kranzelic@erf.unizg.hr (V.K.); 2 The Social Development Research Group, School of Social Work, University of Washington, Seattle, WA 98195, USA; skinnm@uw.edu

**Keywords:** school attachment, school commitment, family communication, family satisfaction, gambling, violence, substance use, sexual risk behavior, youth

## Abstract

The aim of this study was to examine cross-sectional associations of protective factors within a family and school context with adolescent risk behaviors. The study was conducted among adolescents (*n* = 9682) from five cities in Croatia. Mean age of participants was 16.2 years (*SD* = 1.2), and 52.5% were female. Multigroup structural equation modeling was used to examine relations between school attachment, school commitment, family communication, and family satisfaction with gambling, substance use, violence, and sexual risk behavior. Data analyses were conducted in two sets, the first using the full sample, and the second using a subsample (excluding Zagreb) for which there was data on sexual risk behavior. In the first model, school attachment was negatively associated with gambling and violence, while school commitment was negatively associated with students’ gambling, substance use, and violence. Gambling was also associated with family satisfaction in this model. Results from the subsample model were similar with regards to school and family factors associated with gambling, substance use, and violence, with a few exceptions. In this model, family protective factors were found not to be significantly related with any risk behavior. These study results emphasize the importance of strengthening school protective factors, school attachment, and school commitment in preventing risk behaviors in adolescents.

## 1. Introduction

Adolescence is one of the most important developmental periods, during which a person matures physically, cognitively, mentally, emotionally, and socially [1]. Vulnerability and specific characteristics of their age group place adolescents at higher risk when they engage in behaviors such as sexual risk, peer violence, tobacco, alcohol, illicit substance use, and gambling. Youth gambling is a frequent risk behaviour of adolescents in Croatia, where the study presented in this paper was conducted. It was found that 72.9% of Croatian adolescents had at least one experience with gambling in their lifetime [2]. A recent meta-analysis of 44 studies on youth gambling [3] showed that 0.2–12.3% of young people met the criteria for problematic gambling. Compared with other European countries, the highest rate of youth gambling (12.3%) was found in Croatia. At the time of this work, more than three quarters of 16-year-old adolescents (79%) had consumed alcohol in the last year, with almost half (47%) of those having done so in the last month [4] Regarding the consumption of illegal drugs, Croatia was above the European average, with 21% of adolescents stating that they had consumed drugs at least once. High-risk marijuana use among students was also above the European average, with 4.7% of students at risk of developing cannabis-related problems [4]. The results related to the use of inhalants showed that Croatia was at the very top of European countries, with 15% of students having consumed such products at least once. Furthermore, Croatian high school students were at the very top among the European countries in cigarette smoking (29% have smoked in the last month) [4]. A national study on the prevalence of peer violence [5] showed that 8.3% of male and 7.7% of female 15-year-old students were abused by their peers at least twice in the past few months, while 9.4% of male and 4.4% of female students were violent towards their peers at least twice in the past few months. Compared to other European countries, the rate at which 15-year-olds were abused by their peers was similar to those of other countries. A study of sexual risk behavior of adolescents showed that 20.9% of high school students from Croatia had sexual intercourse under the influence of alcohol, and 5.2% under the influence of drugs. As for the use of condoms, 29.9% of sexually active high school students used a condom during their last sexual intercourse, while 59.6% of students used condoms often or always [6].

Although not all young people engage in these behaviors, there is a high correlation between risk behaviors in adolescents and many undesirable outcomes, including morbidity and mortality [7,8].

A significant number of studies have focused on understanding the influence of individual, family, school, and environmental characteristics on adolescent behavioral outcomes [9,10,11]. These studies were predominantly focused on understanding risk factors that contribute to the occurrence of risk behaviors in young people, while protective factors were less often a focal point. Since interest in understanding positive development in young people has grown [12,13,14,15], protective factors have become the focus of more intensive investigation [16,17,18]. Characteristics within the individual or conditions in the family, school, or environment help someone cope successfully with life challenges. They are instrumental in healthy development; they build resiliency, skills and connections, and they reduce the likelihood of problem behavior, either directly or by mediating or moderating the effect of exposure to risk factors [19].

Parents and guardians play an important role in guiding the attitudes and behavior of adolescents [20]. In a systematic review, it was found that parenting practices, especially family communication and parental monitoring, prevented drug initiation and delayed alcohol initiation and sexual debut in adolescents [21]. Nurturing, open, and effective communication between parents and their children correlated to less engagement of adolescents in substance use [22,23]. A good parent–child relationship [24], openness in parent–child communication [25], and a continuous, direct communication style [22] have been shown reduce adolescent substance use. Studies have shown that maternal and paternal communication about sexuality are related to better sexual knowledge in adolescents [26], while parental monitoring and rules were found to be a significant protective factor in early sexual initiation [27]. Preadolescents’ perceptions of parent–child communication and their levels of school-based aggressive behavior were found to be negatively correlated [28]. Positive relationships with parents have been found to be a protective mechanism in development of gambling in adolescents [29,30], as have family cohesion [31], parental care [32], and parental involvement [29]. In earlier studies, a decreased level of parental trust and communication was linked with an increase in gambling behavior [33].

The relationship between adolescents’ satisfaction with their family life and risk behaviors among adolescents has not been well researched. However, studies have associated family life satisfaction with more positive developmental outcomes in adolescents [34,35]. Despite the demonstrated relationship between family life satisfaction and adolescent developmental outcomes, there was no clear evidence of a relationship between family life satisfaction and substance use among adolescents. Earlier studies conducted with high school students and adolescents suggested that there was no relationship between substance abuse and individual characteristics of adolescent self-esteem or family life satisfaction within the pathway model [36,37].

In addition to family protective factors, school environment can also play a significant role in preventing adolescent risk behaviors. School connectedness was found to be related to lower involvement in excessive gambling, substance use, and multiple risk-taking activities [38]. Adolescents who were less connected to school were involved in gambling activities to a higher extent [31] and experienced more socio-emotional harm [39]. In one study [3], negative associations were found between school achievement and problem gambling, including truancy, difficulty in school, decreased academic performance, and school dropout. Many studies found that school attachment and school commitment were negatively associated with substance use [40,41,42]. Better relationships with peers, a stronger attachment to school, and higher academic achievement were negatively associated with smoking [43]. Adolescents who had strong bonds to school were less likely to report nonmedical use of prescription drugs [44]. Promoting school attachment, particularly during the middle school years, can be protective in preventing early substance use [45]. School bonding degree reported by students, a positive relationship with school, school satisfaction, and belonging were found to be negatively associated with violent behavior [46,47,48]. School disengagement—measured on standardized test scores, attendance, failing core courses, suspensions, and grade retention—was found to be related to higher levels of violent behavior during adolescence and early adulthood [49]. In their study with adolescents, Peterson and colleagues [50] found that reduced sexual risk behavior was associated with several school protective factors such as school commitment, belonging, relationships, and participation. Good relationships with teachers and stronger student-level commitment were significantly associated with decreased odds of sexual debut at the 24-month follow-up. In this study, baseline students who reported higher commitment to learning were less likely to report sexual debut at the 36-month follow-up. Students who had stronger relationships with teachers were less likely to report sexual debut at 36 months. They were also less likely to report a failure to use contraception if they were sexually active.

The aim of this study was to examine the cross-sectional associations of family (family communication and family satisfaction) and school protective factors (school attachment, school commitment) with several different adolescent risk behaviors, including substance use, violence, gambling, and sexual risk behavior. In this and some previous studies, family satisfaction was operationalized as the satisfaction with the level of family functioning, i.e., the degree of student satisfaction with the level of support they receive, the ways in which family problems are solved, the quality of time spent together, and the degree of independence within the family [51]. School attachment referred to students’ emotional attachment to school, while school commitment reflected students’ efforts invested and success achieved in school tasks [52,53].

## 2. Materials and Methods

### 2.1. Participants

This cross-sectional survey-based research included a total of 9682 high-school students from five Croatian cities: Zagreb, Pula, Osijek, Split, and Varaždin. In all cities except Zagreb, the intention was to include a sample of 25% of the total high-school population in that city. In Pula, Osijek, Split, and Varaždin, all schools that were willing to participate in the research were included in the research. A total of 25% of high school students was achieved in Varaždin and Osijek, while Split and Pula had lower percentage of involved students than planned.

The target subsample for the city of Zagreb, the capital of Croatia, was 15% of the high-school population, given the large number of students in Zagreb’s high-schools. This subsample of Zagreb was stratified according to three types of high school programs: general preparatory grammar education, 3-year educational schools, and 4-year educational schools. The number of schools in each stratum was calculated based on the total number of students in a particular program, while each school was selected according to the school size, average school achievement, location, and the ratio of boys and girls in a particular school, in order to achieve a representative sample of the Zagreb high school student population.

Altogether, 77 schools participated in the study. Study participants were aged between 14–19 years (*M* = 16.2, *SD* = 1.2), with 52.5% (*n* = 5087) participants being female and 4.5% of participants (*n* = 456) not reporting gender. Additionally, 26.5% were enrolled in a professional 3-year education program, 49.9% in a professional 4-year education program, and 23.6% in a general education program. Sociological demographic characteristics of participants are presented in Table 1.

### 2.2. Measures

The instrument consisted of scales measuring school and family related protective factors, and different risk behaviors of adolescents previously validated in several Croatian studies [54,55,56].

#### 2.2.1. School Protective Factors

School attachment, i.e., students’ emotional attachment to teachers and school, was assessed using 10 items of the School Bonding Scale [54] (α = 0.89). Examples of the items included: “I like going to school.” and “I have good communication with my teachers.” All items were endorsed using a four-point rating scale, with response options ranging from “never” to “very often”.

School Commitment, i.e., students’ efforts invested and success achieved in school tasks, was measured using 7 items of the School Bonding Scale [54] (α = 0.89). This scale was also validated in previous studies with Croatian students. Examples of the items included: “I study regularly.” and “I strive to be a better student.” Responses were given on a four-point rating scale, with response options ranging from “never” to “very often”.

#### 2.2.2. Family Protective Factors

Family Satisfaction was assessed with the Family Satisfaction Scale [55,57]. Examples of this 10-item scale (α = 0.94) related to family life included: “How satisfied are you with your family’s ability to share positive experiences?” and “How satisfied are you with the amount of time you spend together as a family?” Responses were given on a five-point rating scale, with response options ranging from “very dissatisfied” to “very satisfied”.

The Family Communication Scale [55,57], a 10-item scale (α = 0.93), was used to assess communication within the family. Examples of the items included: “Family members can calmly discuss problems with each other.” and “Family members express their true feelings to each other.” For each statement, responses were given on a five-point rating scale, with response options ranging from “strongly disagree” to “completely agree”.

#### 2.2.3. Risk Behaviors

Each indicator of risk behavior was dichotomized into ‘no risk’ and ‘risky’ categories. Gambling activities were assessed with the Gambling Activity Questionnaire [58], which contained questions on the frequency of playing the six most available games (sports betting, betting on virtual races, slot machines, lotto, scratch card games, roulette or any other casino games). The task of the participants was to indicate for each game how often they participated in it in the last 3 months. The response options ranged from 1 to 6 with numbers indicating the following frequencies: 1 = never, 2 = 1–2 times in the past three months, 3 = 1–2 times a month, 4 = 1 per week, 5 = several times a week and 6 = every day. Values ≥ 2 were considered risky levels of gambling activity.

Substance use was assessed with the CTC Youth Survey [56,59] subscale, which contained nine questions on the frequency of using substances during the last 30 days, including drinking beer, wine and/or hard liquors, using marijuana, LSD or other psychedelics, cocaine or crack, ecstasy, amphetamines (speed), prescription medicine without doctor’s orders, and sniffing glue or inhaling gasses or sprays in order to get high. The response options ranged from 1 to 5 with numbers indicating the following frequencies: 1 = never, 2 = 1–2 times, 3 = once a week, 4 = few times a week, 5 = every day. Values ≥ 2 were considered risky levels of substance use.

Violent behavior was also assessed with the CTC Youth Survey [56,59] items “How many times in the past 4 weeks have you taken part in a fight?” and “How many times in the past 4 weeks have you been hurt or have you had to ask for doctor’s help for participating in a fight?”. The response options ranged from 1 to 5, with numbers indicating the following frequencies: 1 = never, 2 = once, 3 = several times, 4 = once a week, 5 = every day. Values ≥ 2 were considered risky levels of violent behavior.

Sexual risk behavior was assessed with two questions. One was focused on assessing frequency of condom use during sexual intercourse: “If you had sexual intercourse, how often do you use condoms?”. Responses ranged from 1 to 4 with numbers indicating the following: 1 = always, 2 = occasionally, 3 = I do not use it, 4 = I do not use it because they reduce satisfaction. Values ≥ 2 were considered risky levels of this behavior. The second question was focused on assessing the number of sexual partners, where having 2 or more partners was considered risky sexual behavior.

### 2.3. Procedure

The study was approved by the Ethical Committee of the Faculty of Education and Rehabilitation Sciences, University of Zagreb, and Croatian Ministry of Science and Education. Local Offices for Education within city or regional levels also approved the study. This study was conducted during October 2018 in Zagreb and in the spring of 2019 in other cities. Before conducting the study, the study team organized meetings with school headmasters and school counselors in each of the five sites to obtain their consent and support. Participation in the study was anonymous and voluntary. Study participants who were 14 and older could give their written consent autonomously, according to the Code of Ethics for Research with Children [60]. The consent contained information on the research and its objectives, ways of approaching the data, the rights of participants, and possible risks. Furthermore, parents received letters in which study aims were described. Students completed the survey during school hours in their classrooms. The research was conducted by researchers and specially trained research assistants, undergraduate and graduate students.

### 2.4. Data Analysis

**Figure 1:**
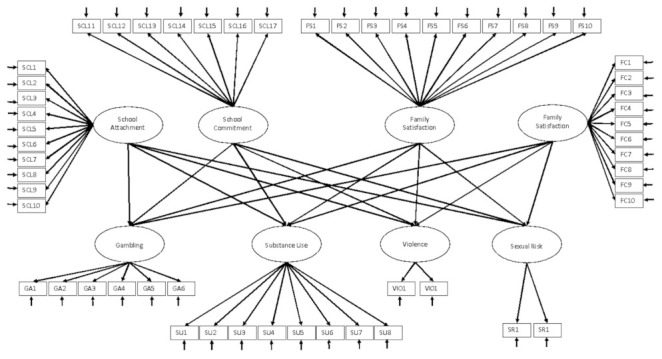
Structural equation model of the association between family and school protective factors with gambling, substance use, violence, and sexual risk behavior.

Structural equation modeling (SEM) [61,62] was employed to test for the associations between school and family factors and risk behaviors. SEM is particularly suited to this task because it uses latent variables based on multiple observed variables for each construct. In this way, all available indicators of each construct can be used simultaneously in a specific factor structure, and associations between constructs are estimated in the context of these specified factors. This affords the advantage of being able to examine the validity of both the measurement model and the structural model in the same analysis. It also avoids problems associated with creating scale summary scores, which obscure relevant psychometric properties. Missing data were handled, in the context of model estimation, by using full information maximum likelihood estimation (FIML) in Mplus version 8.4 [63]. Figure 1 presents the hypothesized model, in which school protective factor (school attachment and school commitment) and family protective factor (family communication and family satisfaction) latent variables were associated with gambling, substance use, violence, and sexual risk behavior. Rectangles represent observed variables, while circles represent latent constructs.

Data analyses were conducted in two sets: first, using the full sample (female *n* = 5086, male *n* = 4594), and then on the subsample (excluding Zagreb), for which there was data on sexual risk behavior (female *n* = 2760, male *n* = 2469). In each case, the measurement models were tested first, and structural models were estimated based on the successful estimation of the measurement models. Multigroup models were specified a priori, allowing all parameters to vary between groups (female, male). Model fit was assessed using model root-mean square error of approximation (RMSEA) and standardized root mean square residual (SRMR), as well as a test of close fit (the probability that RMSEA is below 0.05). Comparative fit was evaluated using the comparative fit index (CFI) and Tucker–Lewis index (TLI). Based on previously published results [64], the measurement models were specified with all of the latent constructs, allowing covariances and residual covariances among several scale variables to be freely estimated.

## 3. Results

Using the full sample, the initial measurement model, including seven latent variables (school attachment and commitment, family communication and satisfaction, gambling, substance use, and violence) achieved adequate fit (RMSEA = 0.04; probability RMSEA < 0.05 = 1.00; SRMR = 0.04; CFI = 0.94; TLI = 0.94). Based on this measurement model, the structural model was estimated and found to have adequate fit (RMSEA = 0.04, probability RMSEA < 0.05 = 1.00, SRMR = 0.04) and comparative fit indices (CFI = 0.94, TLI = 0.94).

Results of the model using the full sample are presented in Table 2 including standardized effect sizes, *p*-values, and upper and lower bounds of their 95% confidence intervals (CI).

For both females and males, school factors had more significant negative associations with outcomes than family factors.

In both groups, school attachment was significantly associated with gambling. Among females, each standard deviation (SD) increase in school attachment was associated with a 06 SD decrease in gambling. Among males, each SD increase was associated with a 12 SD decrease in gambling. Among males, there was also a 13 SD decrease in violence.

School commitment was significantly associated with gambling, substance use, and violence for both groups. Among females, each SD increase in school commitment was associated with an 18 SD decrease in gambling, a 13 SD decrease in substance use, and a 12 SD decrease in violence. Among males, each SD increase in school commitment was associated with a 23 SD decrease in gambling, a 9 SD decrease in substance use, and an 11 SD decrease in violence.

No significant associations were observed between family communication and any of the outcomes for either group. Family satisfaction was positively associated with gambling among males, such that each SD increase in family satisfaction was associated with an 08 SD increase in gambling.

Test of the measurement model using the subsample (excluding Zagreb) and including eight latent constructs (school attachment and commitment, family communication and satisfaction, gambling, substance use, violence, and sexual risk behavior) achieved adequate fit (RMSEA = 0.04; probability RMSEA < 0.05 = 1.00; SRMR = 0.05; CFI = 0.92; TLI = 0.92). Results for the final model using the subsample are presented in Table 3.

Results were similar with regards to school and family factors associated with gambling, substance use, and violence, with a few exceptions. Among males, a significant association was observed between school attachment and substance use, such that for each SD increase in attachment there was a 09 SD decrease in substance use. The positive relationship observed in the full sample between family satisfaction and gambling among adolescent males was not significant in this subsample. Significant associations were observed between sexual risk behavior and school factors. Among males, each SD increase in school attachment was associated with an 11 SD decrease in sexual risk behavior. Each SD increase in school commitment was associated with an 18 SD decrease in sexual risk behavior among males and a 14 SD decrease in sexual risk behavior among females.

## 4. Discussion

The results of this study showed that school protective factors had more significant negative associations with the observed risk behaviors than family factors for both females and males.

In the first model, which included data from the full sample, school attachment was significantly negatively associated only with gambling, and this relationship was significant for both genders. This finding was in accordance with several previous studies in which school connectedness was identified as protective mechanisms in relation to youth gambling problems [31,33,65,66,67]. Among males, school attachment was significantly negatively associated with violent behavior. Earlier studies have found that relationship as well, albeit for both genders [46,47,48]. The National Longitudinal Study of Adolescent Health (Add Health) [68] showed that girls’ levels of attachment to school were higher in middle school, while boys’ levels were higher in high school. Adolescent males were more likely to engage in physical fights than females [69]. It would seem that school attachment plays an important role in preventing this behavior that should be studied more.

In this study, school commitment was significant negatively associated with gambling, substance use, and violence for both groups. One recent study [39] showed that students who had poor school performance and academic achievement gambled more frequently. Gambling activities could expose adolescents to antisocial peer groups, which in turn might influence school engagement and school performance, either directly or through the increase of behavioral and social problems [70]. In previous studies, low school commitment was strongly associated with illicit drug use [71] and smoking [72], while adolescents who dropped out of school were at significantly increased risk for using cigarettes, marijuana, and alcohol [73]. In addition, increased alcohol use was associated with academic difficulties, lower grade point average, and increased risk of dropping out of school [74]. The association between school disengagement and violent behavior was also confirmed in earlier studies with adolescents [49].

Regarding the observed family protective factors, no significant associations were found between family communication and any of the risk behaviors for either gender. Previous studies were predominantly focused on examining the relationship between family communication and alcohol and substance use among young people. These studies showed that the quality of family communication played a significant role in decreasing substance use [23,28] and binge drinking [75,76] among youth. However, these studies were conducted with preadolescents whose age was lower than the age of a sample in our study. A study conducted with adolescents whose mean age was closer to the one in our sample (mean age = 15.1 years) showed that communication with parents did not predict alcohol or drug use, but was associated with academic motivation and peers, which were associated with substance use. According to the authors of that study [77], communication with parents may have an indirect role in alcohol and drug use, by influencing adolescents’ academic motivation and choice of friends. During adolescence, young people are developing autonomy from their parents so their peers become a significant source of social and emotional support [78], which makes peer relationships very influential during this life period.

Interestingly, family satisfaction was positively associated with gambling among males, indicating that adolescents who were more satisfied with their families were more frequently involved in gambling activities. Previous studies have shown that poor parental supervision has been linked to higher involvement in gambling behavior and, consequently, larger problems related to gambling [32,79]. A study that aimed to assess the relationships between parenting styles, parents’ attitudes toward gambling, and frequency of parents’ gambling and frequency of their sons’ gambling showed that a certain number of male adolescents participated in gambling activities together with their fathers [80]. The same study showed that there was a significant relationship between permissive parenting style and adolescent gambling. For some adolescents, having permissive parents might increase their satisfaction with their family life since it gives them a lot of freedom. Further research is needed to understand this relationship between family life satisfaction and gambling activities of adolescents.

Results for the subsample model were similar with regards to school and family factors associated with gambling, substance use, and violence with a few exceptions. Among males, a significant negative association was observed between school attachment and substance use. The Canadian International Youth Survey (IYS) [81] showed that youth with higher levels of school attachment were less likely to use substances in the past month than youth with lower levels of school attachment.

The positive relationship observed in the full sample between family satisfaction and gambling among adolescent males was not significant in this subsample.

Significant negative associations were observed between both school factors and sexual risk behavior. School attachment was negatively associated with sexual risk behavior only among males, while school commitment was negatively associated with sexual risk behavior among both males and females. The INCLUSIVE study from England [50] included 24 and 36 months of follow-up in order to examine schools’ effects on students’ risky sexual behavior, sexual debut, and use of contraception. At 36-month follow-up, baseline students who reported stronger relationships with teachers were less likely to report sexual debut at 36 months, and less likely to report a failure to use contraception if they were sexually active. Associations involving participation and contraception use were largely nonsignificant in the described study. More research is needed to deepen our understanding of the relationships between school factors and sexual risk behavior.

Family protective factors were found not to be significantly related with the observed risk behaviors in the final sample model.

There were several limitations in this study. First, because of the cross-sectional study design, the causal relations among observed protective factors and gambling, violence, substance use, and sexual risk behavior could not be examined. In order to examine the directionality of these relations, future longitudinal studies are needed. Additionally, the data were collected exclusively using the method of self-assessment. As some questions were focused on assessing participation in risk behaviors, it is possible that some adolescents gave socially desirable answers, given the sensitivity of the question. Additional assessments gained from teachers, parents and/or peers might have provided more accurate insights. The study measures were extracted from a comprehensive set of measures used with students which measured many different constructs and behaviors. For that reason, sexual risk behavior and violence were each assessed with only two items. Having comprehensive scales to measure these behaviors would make the findings of this study stronger. It is important to note that this research was conducted before the beginning of the COVID-19 virus pandemic, which significantly affected the modalities of teaching and social interaction of high school students. It is very likely that these circumstances could have influenced family and school context characteristics. The occurrence of risk behaviors of adolescents would be worth exploring in post-COVID-19 circumstances.

## 5. Conclusions

The impact of a family context on the developmental path of an individual is unquestionable. However, during adolescence, peers and a school environment have a significant socializing influence on adolescent behavior. Peer relationships serve as a bridge as adolescents move away from their parents and toward independent adult functioning [82]. In schools, adolescents are provided with opportunities to practice and explore their relationships with others. Furthermore, schools have a power to promote student wellbeing and positive development by increasing students’ commitment to learning and sense of belonging through a focus on students’ needs and engagement and the development of their practical reasoning abilities and affiliations. School connectedness, engagement in learning, investment in the school community, and a positive school climate help to neutralize the risks associated with risk behaviors and related negative outcomes [44].

This study’s results emphasized the importance of investing into specific school protective factors, e.g., school attachment and school commitment, in preventing different risk behaviors in adolescents. School attachment, i.e., students’ emotional attachment to teachers and school, was negatively associated with gambling for both genders and with violence for male adolescents. In the subsample model, school attachment was also negatively associated with sexual risk behavior for males. In this study, school commitment, i.e., students’ efforts invested and success achieved in school tasks, was negatively associated with gambling, substance use, and violence for both genders. Centers for Disease Control and Prevention [73] suggested several strategies to promote adolescents’ school connectedness, i.e., the belief (by students) that adults and peers in the school care about their learning as well as about them as individuals. These were: (1) creating decision-making processes that facilitate student, family, and community engagement, academic achievement, and staff empowerment; (2) providing education and opportunities to enable families to be actively involved in children’s’ academic and school life; (3) providing students with the academic, emotional, and social skills necessary to be actively engaged in school; (4) using effective classroom management and teaching methods to foster a positive learning environment; (5) providing professional development and support for teachers and other school staff to enable them to meet the diverse cognitive, emotional, and social needs of children and adolescents; (6) creating trusting and caring relationships that promote open communication among administrators, teachers, staff, students, families, and communities.

Due to the significant impact schools have on adolescents; school principals and teachers should be continuously supported through national and local policies and strategies in developing relevant knowledge and skills for science-based prevention of risk behaviors. Effective approaches to prevention within a school context should include both, minimizing risk factors, and strengthening protective factors and conditions that promote positive youth development.

## Figures and Tables

**Table 1:** Sociological demographic characteristics by data collection site.

	Zagreb	Split	Osijek	Pula	Varaždin	Total
*n* (%)	4453 (46.0)	1239 (12.8)	1676 (17.3)	704 (7.3)	1610 (16.6)	9682 (100)
Age, *n* (%) ^1^						
14	158 (3.6)	0	51 (3.0)	15 (2.1)	73 (4.5)	297 (3.1)
15	1352 (30.4)	256 (20.7)	469 (28.0)	216 (30.7)	450 (28.0)	2743 (28.4)
16	1189 (26.8)	350 (28.2)	433 (25.9)	163 (23.2)	387 (24.0)	2522 (26.1)
17	1083 (24.4)	341 (27.5)	427 (25.5)	163 (23.2)	407 (25.3)	2421 (25.0)
18	621 (14.0)	247 (19.9)	280 (16.7)	138 (19.6)	268 (16.6)	1554 (16.1)
19	39 (0.9)	45 (3.6)	14 (0.8)	9 (1.3)	25 (1.6)	132 (1.4)
Gender, *n* (%)						
Female	2327(52.3)	670 (54.1)	901(53.8)	427 (60.7)	762 (47.3)	5087(52.5)
Male	2126 (47.7)	569 (45.9)	775 (46.2)	277 (39.3)	848 (52.7)	4595 (47.5)

^1^ 11 participants from Zagreb and 2 participants from Osijek were missing age information.

**Table 2:** Results of structural equations examining the association of family and school protective factors and family satisfaction with female and male students gambling, substance use, and violence.

	Female				Male			
	*n* = 5086				*n* = 4594			
	*β*	*p*-Value	95% CI		*β*	*p*-Value	95% CI	
			Lower	Upper			Lower	Upper
***School Attachment***								
**Gambling**	**−0.06**	**0.005**	**−0.11**	**−0.02**	**−0.12**	**0.000**	**−0.16**	**−0.08**
**Substance Use**	0.00	0.981	−0.04	0.04	−0.08	0.001	−0.12	−0.03
**Violence**	−0.04	0.097	−0.08	0.01	**−0.13**	**0.000**	**−0.19**	**−0.08**
***School Commitment***								
**Gambling**	**−0.18**	**0.000**	**−0.22**	**−0.13**	**−0.23**	**0.000**	**−0.27**	**−0.19**
**Substance Use**	**−0.13**	**0.000**	**−0.17**	**−0.09**	**−0.09**	**0.000**	**−0.13**	**−0.05**
**Violence**	**−0.12**	**0.000**	**−0.17**	**−0.08**	**−0.11**	**0.000**	**−0.17**	**−0.05**
***Family Communication***								
**Gambling**	0.06	0.313	−0.05	0.17	0.02	0.518	−0.04	0.08
**Substance Use**	−0.01	0.891	−0.11	0.09	−0.03	0.375	−0.09	0.03
**Violence**	−0.09	0.095	−0.20	0.02	−0.08	0.068	−0.16	0.01
***Family Satisfaction***								
**Gambling**	−0.04	0.530	−0.14	0.07	**0.08**	**0.017**	**0.01**	**0.14**
**Substance Use**	−0.09	0.068	−0.19	0.01	−0.02	0.466	−0.08	0.04
**Violence**	0.02	0.688	−0.09	0.13	0.03	0.442	−0.05	0.11

Note: boldface indicates statistically significant results using a significance level of α = 0.05. β, standardized model coefficient; CI, 95% confidence interval.

**Table 3:** Results of structural equations examining the association of family and school protective factors with female and male students’ gambling, substance use, violence, and sexual risk behavior in available subsample.

	Female				Male			
	*n* = 2760				*n* = 2469			
	*β*	*p*-Value	95% CI		*β*	*p*-Value	95% CI	
			Lower	Upper			Lower	Upper
***School Attachment***								
**Gambling**	**−0.09**	**0.003**	**−0.15**	**−0.03**	**−0.11**	**0.000**	**−0.17**	**−0.05**
**Substance Use**	0.01	0.779	−0.05	0.06	**−0.09**	**0.002**	**−0.15**	**−0.03**
**Violence**	−0.05	0.079	−0.11	0.01	−0.07	0.070	−0.15	0.01
**Sexual Risk**	−0.01	0.728	−0.07	0.05	**−0.11**	**0.002**	**−0.18**	**−0.04**
***School Commitment***								
**Gambling**	**−0.12**	**0.000**	**−0.18**	**−0.06**	**−0.26**	**0.000**	**−0.32**	**−0.20**
**Substance Use**	**−0.13**	**0.000**	**−0.18**	**−0.07**	**−0.10**	**0.001**	**−0.16**	**−0.04**
**Violence**	**−0.09**	**0.002**	**−0.15**	**−0.03**	**−0.12**	**0.003**	**−0.20**	**−0.04**
**Sexual Risk**	**−0.14**	**0.000**	**−0.20**	**−0.08**	**−0.18**	**0.000**	**−0.24**	**−0.11**
***Family Communication***								
**Gambling**	0.00	0.970	−0.13	0.13	0.03	0.444	−0.05	0.12
**Substance Use**	0.00	0.979	−0.12	0.12	−0.04	0.312	−0.13	0.04
**Violence**	−0.11	0.103	−0.24	0.02	−0.05	0.383	−0.16	0.06
**Sexual Risk**	0.02	0.719	−0.11	0.16	0.10	0.059	0.00	0.20
***Family Satisfaction***								
**Gambling**	0.04	0.560	−0.09	0.16	0.07	0.110	−0.02	0.15
**Substance Use**	−0.12	0.054	−0.23	0.00	0.02	0.701	−0.07	0.10
**Violence**	0.05	0.449	−0.08	0.17	−0.01	0.843	−0.12	0.10
**Sexual Risk**	−0.12	0.069	−0.25	0.01	−0.09	0.083	−0.18	0.01

Note: boldface indicates statistically significant results using a significance level of α = 0.05. β, standardized model coefficient; CI, 95% confidence interval.

## Data Availability

The data presented in this study is available on request from the corresponding author. Due to privacy issues, the data is not publicly available.
